# Melatonin alleviates lipopolysaccharide‐compromised integrity of blood–brain barrier through activating AMP‐activated protein kinase in old mice

**DOI:** 10.1111/acel.12572

**Published:** 2017-02-03

**Authors:** Xiaona Wang, Gai‐Xiu Xue, Wen‐Cao Liu, Hui Shu, Mengwei Wang, Yanyun Sun, Xiaojing Liu, Yi Eve Sun, Chun‐Feng Liu, Jie Liu, Wenlan Liu, Xinchun Jin

**Affiliations:** ^1^ Jiangsu Key Laboratory of Translational Research and Therapy for Neuro‐Psycho‐Diseases and Institute of NeuroscienceThe Second Affiliated Hospital of Soochow UniversitySuzhou215004China; ^2^ Suzhou Municipal HospitalSuzhou215002China; ^3^ Department of EmergencyShanxi Provincial People's HospitalTaiyuan030001China; ^4^ Translational Center for Stem Cell ResearchTongji HospitalStem Cell Research CenterTongji University School of MedicineShanghai200065China; ^5^ Department of Psychiatry and Biobehavioral SciencesDavid Geffen School of MedicineUniversity of California, Los AngelesLos AngelesCA90095USA; ^6^ Department of NeurologyJiangsu Key Laboratory of Translational Research and Therapy for Neuro‐Psycho‐DiseasesThe Second Affiliated Hospital of Soochow UniversitySoochow UniversitySuzhou215004China; ^7^ The Central LaboratoryShenzhen Second People's Hospitalthe First Affiliated Hospital of Shenzhen UniversityShenzhen518035China

**Keywords:** AMPK, blood–brain barrier, lipopolysaccharide, Melatonin, old mice, tight junction protein

## Abstract

Blood–brain barrier (BBB) dysfunction is considered to be an early event in the pathogenesis of a variety of neurological diseases in old patients, and this could occur in old people even when facing common stress. However, the mechanism remains to be defined. In this study, we tested the hypothesis that decreased melatonin levels may account for the BBB disruption in old mice challenged with lipopolysaccharide (LPS), which mimicked the common stress of sepsis. Mice (24–28 months of age) received melatonin (10 mg kg^−1^ day^−1^, intraperitoneally, i.p.) or saline for one week before exposing to LPS (1 mg kg^−1^, i.p.). Evan's blue dye (EB) and immunoglobulin G (IgG) leakage were used to assess BBB permeability. Immunostaining and Western blot were used to detect protein expression and distribution. Our results showed that LPS significantly increased BBB permeability in old mice accompanied by the degradation of tight junction proteins occludin and claudin‐5, suppressed AMP‐activated protein kinase (AMPK) activation, and elevated gp91^phox^ protein expression. Interestingly, administration of melatonin for one week significantly decreased LPS‐induced BBB disruption, AMPK suppression, and gp91^phox^ upregualtion. Moreover, activation of AMPK with metformin significantly inhibited LPS‐induced gp91^phox^ upregualtion in endothelial cells. Taken together, our findings demonstrate that melatonin alleviates LPS‐induced BBB disruption through activating AMPK and inhibiting gp91^phox^ upregulation in old mice.

**Abbreviations**
AMPK: AMP‐activated protein kinase
BBB: blood–brain barrier
EB: Evan's blue dye
IgG: immunoglobulin G
i.p.: intraperitoneally
LPS: lipopolysaccharide
NADPH: nicotinamide adenine dinucleotide phosphate
p‐AMPK: phosphorylated AMP‐activated protein kinase
ROS: reactive oxygen species
TJPs: tight junction proteins

## Introduction

Blood–brain barrier (BBB) dysfunction is an early event in the pathogenesis of a variety of neurological diseases (Rosenberg, 2012), and this could occur when facing various extrinsic or intrinsic stimuli (Weiss *et al*., 2009). Sepsis is a common stress that old people often face (Martin *et al*., 2006), in which lipopolysaccharide (LPS) is released into circulation (Shukla *et al*., 2014), promoting the generation of reactive oxygen species (ROS) in cerebral microvascular endothelial cells and BBB disruption (Seok *et al*., 2013).

Nicotinamide adenine dinucleotide phosphate (NADPH) oxidases is a major source of ROS generation in the brain, which critically contributes to BBB disruption under various CNS disorders (Kahles *et al*., 2007). There are several NADPH oxidase family members, among which gp91^phox^‐containing NADPH oxidase is highly expressed in the cerebral vascular endothelium (Kahles *et al*., 2007). Suppressing gp91^phox^ has been shown to protect mice from a variety of stimuli that promote cerebrovascular dysfunction (Liu *et al*., 2008; Tang *et al*., 2010).

LPS has been shown to induce BBB dysfunction via NADPH oxidase‐derived ROS (Liu *et al*., 2012; Zhao *et al*., 2014). In addition, it could also inhibit the activation of AMP‐activated protein kinase (AMPK), a serine/threonine protein kinase regulating cellular and organismal metabolism (Zhou *et al*., 2014b). When AMPK is phosphorylated at Thr172 (p‐AMPK), the kinase activity of the α subunit increases >100‐fold (Hawley *et al*., 1996). Interestingly, AMPK activation (p‐AMPK) has been shown to attenuate LPS‐induced BBB disruption *in vitro* (Zhao *et al*., 2014) and protect the BBB in diabetes by inhibiting LPS‐enhanced NADPH oxidase expression in brain capillary endothelial cells (Liu *et al*., 2012).

Melatonin, mainly secreted by the pineal gland (Manchester *et al*., 2015), exerts many physiological and biochemical functions when it is released into blood and cerebrospinal fluid (Reiter *et al*., 2014), such as acting as a circadian rhythm regulator, an anti‐inflammatory and immunoregulating molecule, and an oncostatic agent (Manchester *et al*., 2015). Of note, melatonin and its metabolites are known to scavenge a variety of free radicals and reactive oxygen intermediates *in vivo* and *in vitro* (Galano *et al*., 2014; Manchester *et al*., 2015), which may account for its protective effects against LPS toxicity to the brain (Wong *et al*., 2014; Carloni *et al*., 2016), liver (Wang *et al*., 2007), and heart (Lu *et al*., 2015). Moreover, melatonin also shows protective effects against BBB damage resulting from excitotoxicity in neonatal rats (Moretti *et al*., 2015) and transient focal cerebral ischemia in young mice (Chen *et al*., 2006a,b). However, it is not clear whether low melatonin levels contribute to the BBB disruption in old mice whose melatonin levels in serum and pineal gland decline as a result of aging (Hill *et al*., 2013).

In this study, we investigated the effect of melatonin supplementation on LPS‐induced BBB disruption in old mice and found that LPS disrupted the BBB in old mice via downregulating tight junction protein expression via increasing gp91^phox^ expression and inhibiting AMPK activation, and supplementation of melatonin could effectively inhibit the above changes.

## Results

**Figure 1:**
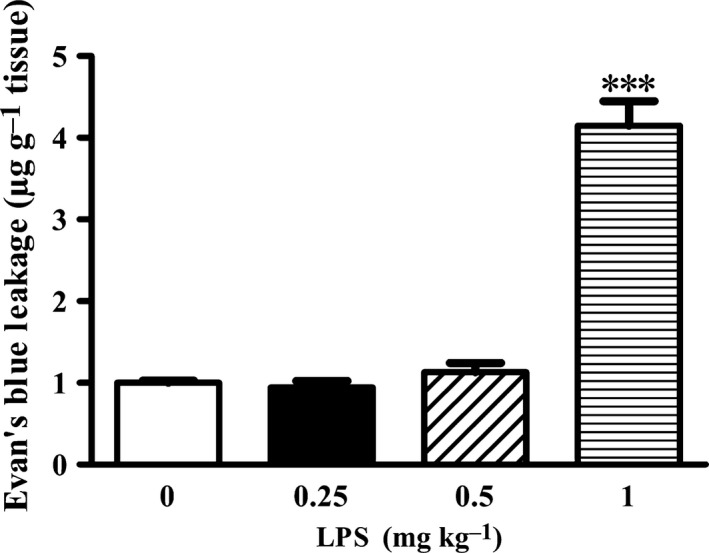
Effect of LPS treatment on BBB integrity in old mice. Quantification of EB leakage showed that LPS at 1 mg kg^−1^, but not at 0.25 or 0.5 mg kg^−1^, significantly increased BBB permeability (***P <* *0.001). *n *= 5–6 for each group. Data were expressed as mean ± SEM.

LPS has been shown to disrupt BBB *in vitro* (Zhao *et al*., 2014) and *in vivo* (young mice) (Zhou *et al*., 2014a). However, its impact on the BBB of old mice remains unknown. To address this question, mice were randomly divided into four groups receiving 0, 0.25, 0.5, and 1 mg kg^−1^ LPS, respectively (Nonaka *et al*., 2004). As shown in Fig. 1, LPS at a dose of 1 mg kg^−1^, but not 0.25 or 0.5 mg kg^−1^, significantly increased the EB leakage in old mice (***P <* *0.001 vs. the Veh group). Therefore, we chose 1 mg kg^−1^ LPS as the treatment for the rest of the study.

**Figure 2:**
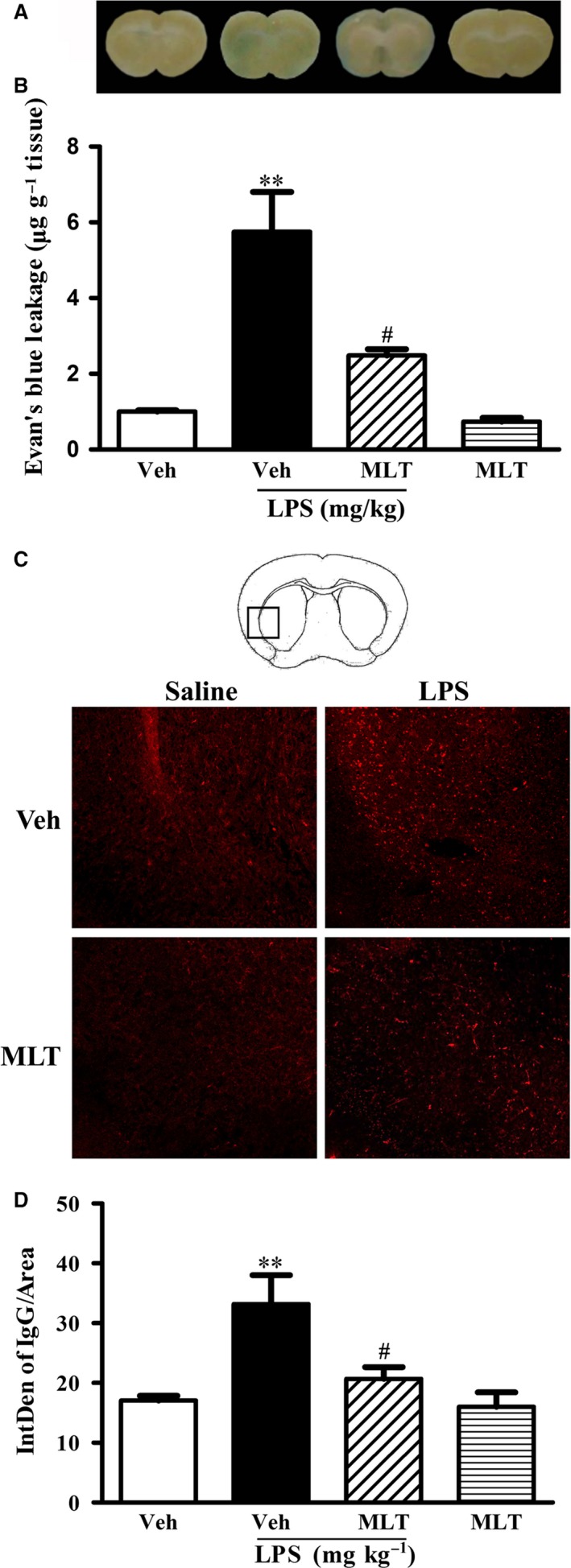
Effect of melatonin on LPS‐induced BBB damage in old mice. (A) Representative brain coronal sections showed EB leakage from vehicle, LPS‐treated, or melatonin‐treated group. (B) Quantification of EB leakage showed that BBB was disrupted in LPS‐treated animals and pretreatment with melatonin significantly alleviated the change (**P <* *0.01 vs. the Veh control, ^#^ *P < *0.05 vs. the Veh+LPS,* n *= 5 for each group). (C) Representative confocal micrographs showed IgG leakage (red fluorescence) in the brain. (D) IgG leakage was quantitated and showed that pretreatment of mice with melatonin reduced LPS‐induced IgG leakage (**P <* *0.01 vs. the Veh control, ^#^ *P *<* *0.05 vs. the Veh+LPS). *n *= 3 for each group. Data were expressed as mean ± SEM.

We next examined the effect of melatonin treatment on LPS‐induced BBB damage in old mice. As shown in Fig. 2A, LPS significantly increased EB leakage (Fig. 2B, **P <* *0.01 vs. the Veh group), and pretreatment with melatonin for one week significantly alleviated LPS‐induced EB leakage in old mice (Fig. 2B, ^#^ *P < *0.05 vs. the Veh+LPS group). As a good complimentary analysis of the BBB permeability, IgG immunostaining was performed to detect IgG extravasation. Consistent with the results of EB leakage, LPS significantly increased IgG leakage (Fig. 2D, **P <* *0.01 vs. the Veh group), which was significantly decreased by pretreatment with melatonin (Fig. 2D, ^#^ *P *<* *0.05 vs. the Veh+LPS group), further supporting that melatonin alleviated LPS‐induced BBB disruption in old mice.

**Figure 3:**
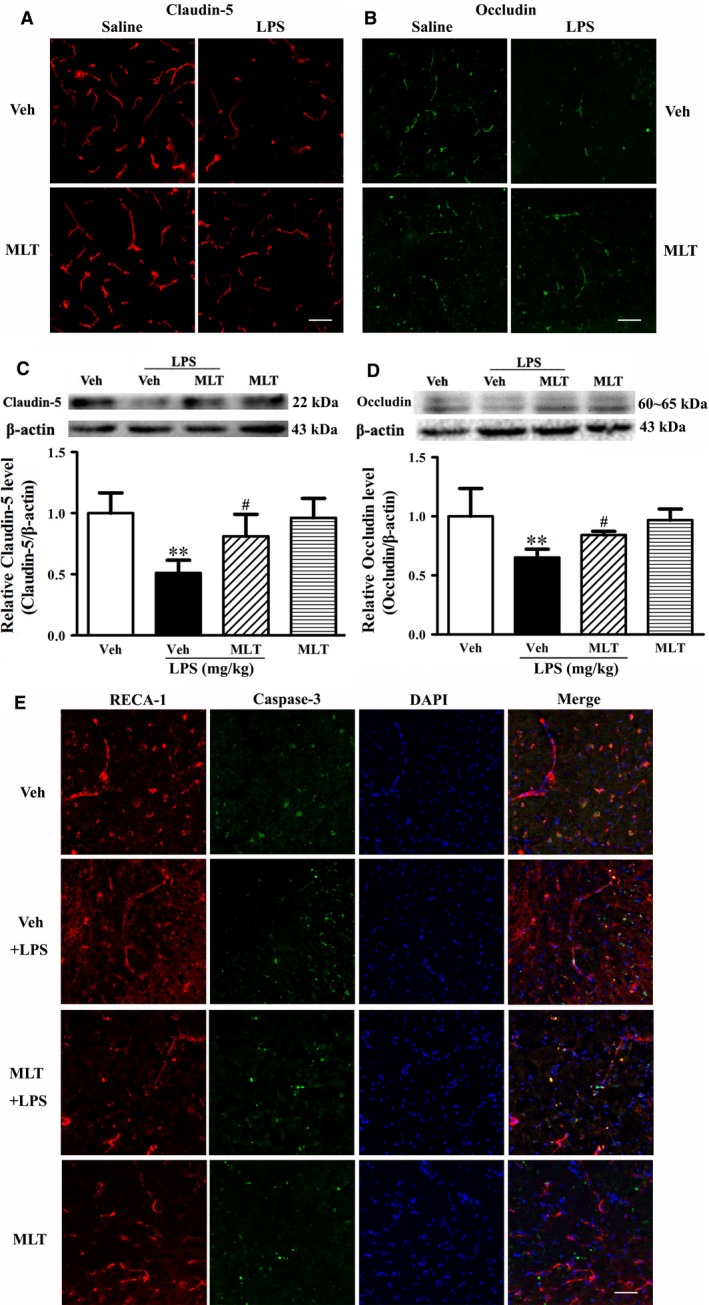
Effect of melatonin on LPS‐induced tight junction protein (TJP) degradation in old mice. Old mice were subjected to the indicated treatment and TJPs claudin‐5 and occludin were analyzed by immunostaining and Western blot. Representative confocal micrographs showed that LPS decreased immunostaining for claudin‐5 (A) and occludin (B), and pretreatment with melatonin ameliorated this change. Experiments were repeated three times with similar results. Western blot analysis for claudin‐5 (C) and occluding (D) confirmed the finding of immunostaining. Representative immunoblots showed the bands of claudin‐5 and occludin (upper panel). The band intensities of occludin and claudin‐5 were quantitated after normalization to the beta actin. LPS induced a significant reduction in the protein levels of claudin‐5 (**P <* *0.01 vs. the Veh) and occludin (*P <* *0.01 vs. the Veh). Treatment with melatonin prevented the reduction in claudin‐5 protein (C, ^#^ *P < *0.05 vs. the Veh+LPS group) and occludin protein (D, ^#^ *P < *0.05 vs. the Veh+LPS group) in LPS‐treated mice. *n *= 4 for each group. Data were expressed as mean ± SEM. Double immunostaining of RECA‐1 and cleaved caspase‐3 showed limited co localization, and melatonin supplementation did not affect this colocalization (E). Scale bar = 50 μm.

Loss or altered distribution of tight junction proteins (TJPs), particularly occludin and claudins, were seen in the compromised BBB following LPS treatment (Zhao *et al*., 2014). Here using immunostaining, our results showed that melatonin decreased LPS‐induced degradation of claudin‐5 (Fig. 3A) and occludin (Fig. 3B) in old mice. The results were further confirmed by Western blot showing that LPS induced a dramatic reduction in total protein levels of claudin‐5 (Fig. 3C, **P <* *0.01 vs. the Veh group) and occludin (Fig. 3D, **P <* *0.01 vs. the Veh group), and these changes were significantly inhibited by melatonin (Fig. 3, ^#^ *P < *0.05 vs. the Veh+LPS group).

Death of endothelial cells of microvessels is a major contributor to the disruption of BBB integrity (Simard *et al*., 2007). In order to determine whether endothelial cell death contributes to LPS‐induced BBB disruption in old mice, double immunostaining of RECA‐1 (marker of endothelial cells) and cleaved caspase‐3 (marker of cell death) was performed. As shown in Fig. 3E, there were limited colocalizations of RECA‐1 with cleaved caspase‐3, and pretreatment with melatonin did not significantly affect this colocalization, suggesting that under our experimental conditions, endothelial cell death was not a major contributor to LPS‐induced BBB damage in old mice.

**Figure 4:**
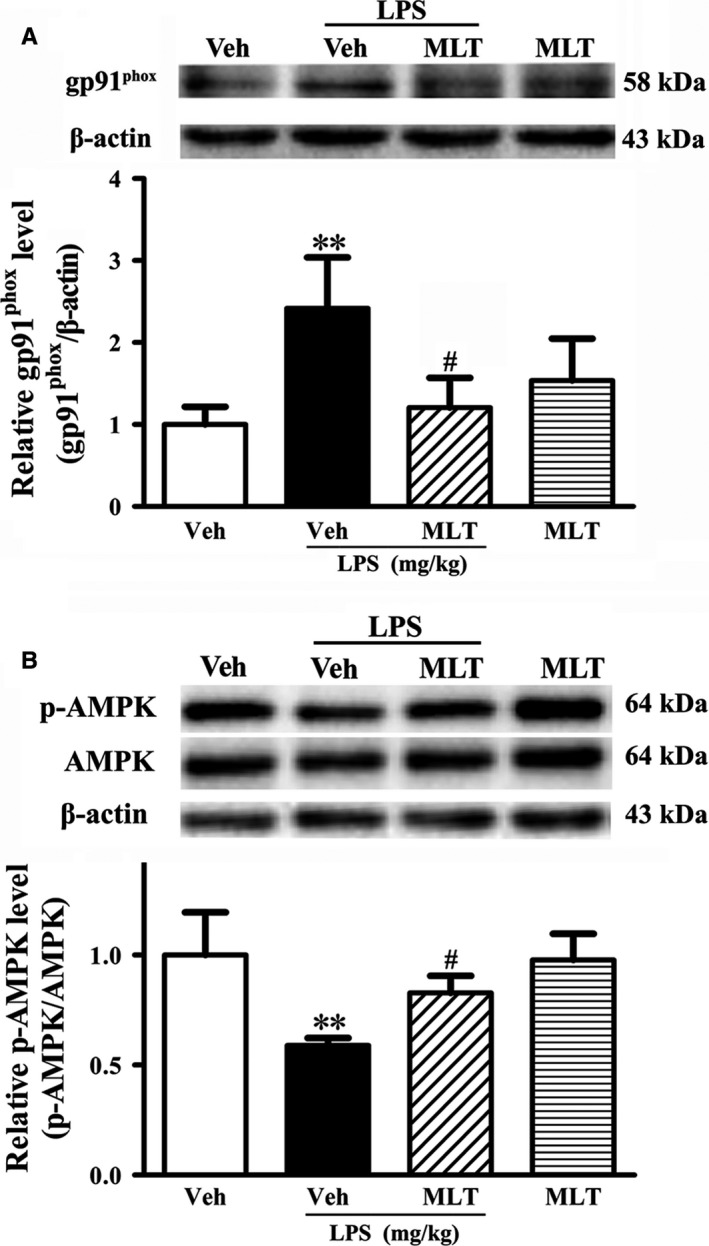
Effect of melatonin on LPS‐induced gp91^phox^ expression upregulation and inhibition of AMPK activation in old mice. The mice were subjected to the indicated treatment before analyzing gp91^phox^ with Western blot. (A) A representative immunoblot showed gp91^phox^ protein expression (upper panel). The band intensity of gp91^phox^ was quantitated after normalization to the beta actin (lower panel) and showed that LPS induced a significant increase in the protein level of gp91^phox^ (**P <* *0.01 vs. the Veh group). Pretreatment with melatonin inhibited LPS‐induced gp91^phox^ upregulation (^#^ *P < *0.05 vs. the Veh+LPS group). *n *= 4 for each group. Data were expressed as mean ± SEM. (B) Representative immunoblots showed the protein bands of p‐AMPK and AMPK (upper panel). The band intensities of p‐AMPK and AMPK were quantitated after normalization to the beta actin (lower panel) and showed that LPS significantly inhibited AMPK activation (**P <* *0.01 vs. the Veh group). Pretreatment with melatonin prevented this inhibition induced by LPS (^#^ *P < *0.05 vs. the Veh+LPS group). *n *= 4 for each group. Data were expressed as mean ± SEM.

We next examined the underlying mechanisms of TJP degradation in old mice challenged by LPS. Given that ROS could disrupt the BBB and gp91^phox^‐containing NADPH oxidase is an important source of ROS in the brain (Liu *et al*., 2008; Tang *et al*., 2010), we examined the expression of gp91^phox^, the catalytic unit of NADPH oxidase. Western blot analysis showed that LPS significantly increased gp91^phox^ protein levels compared with the Veh group (Fig. 4B, **P <* *0.01 vs. the Veh group), and this change was significantly inhibited by melatonin treatment (Fig. 4B, ^#^ *P < *0.05 vs. the Veh+LPS group).

AMPK has been shown to play an important role in maintaining BBB integrity (Liu *et al*., 2012), and LPS has shown an inhibitory effect on AMPK activation (p‐AMPK) (Zhou *et al*., 2014b). Therefore, we examined the role of p‐AMPK in LPS‐induced BBB damage. As shown in Fig. 4, LPS significantly decreased p‐AMPK (Fig. 4D, **P <* *0.01 vs. the Veh group) and melatonin treatment significantly alleviated this effect (Fig. 4D, ^#^ *P < *0.05 vs. the Veh+LPS group).

**Figure 5:**
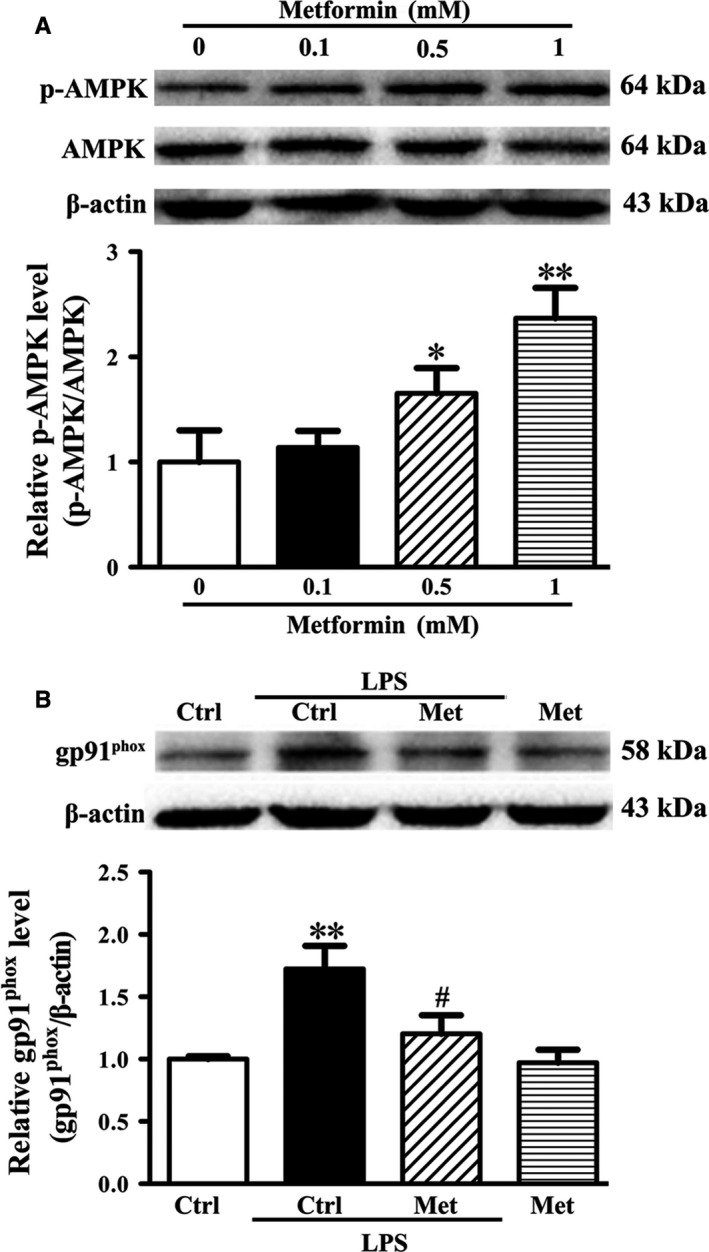
Effect of metformin on LPS‐induced gp91^phox^ expression upregulation in bEND3 cells. bEND3 cells were subjected to the indicated treatment before analyzing AMPK and gp91^phox^ with Western blot. (A) Representative immunoblots revealed the protein band of p‐AMPK and AMPK and metformin at 1 mm significantly activated AMPK (*P <* *0.05,**P <* *0.01 vs. the Veh group). (B) A representative immunoblot showed the protein band of gp91^phox^ (upper panel). The band intensity of gp91^phox^ was quantitated after normalization to the beta actin and showed that LPS treatment significantly upregulated gp91^phox^ expression (**P <* *0.01 vs. the Veh group). Treatment with metformin significantly inhibited this change (^#^ *P < *0.05 vs. the Veh+LPS group). *n *= 6 for each group. Data were expressed as mean ± SEM.

To investigate the relationship between AMPK activation (p‐AMPK) and gp91^phox^ expression after LPS treatment, we performed *in vitro* experiments using cultured brain microvascular endothelial cells (bEND3 cells) and metformin, the AMPK activator (Takata *et al*., 2013). Firstly, we conducted experiments to identify the optimal dose of metformin for the activation of AMPK in endothelial cells and found that incubation of endothelial cells with 1 mm metformin significantly activated AMPK (Fig. 5A,*P <* *0.05, **P <* *0.01 vs. the Veh group). Next, bEND3 cells were treated with or without metformin for 8 h before exposing to 1 μg mL^−1^ LPS for additional 16 h (Zhao *et al*., 2014) before analyzing gp91^phox^ expression by Western blot. As shown in Fig. 5B, LPS significantly upregulated gp91^phox^ expression (**P <* *0.01 vs. the Veh group) and metformin pretreatment significantly decreased LPS‐induced gp91^phox^ expression (^#^ *P < *0.05 vs. the Veh+LPS group), suggesting that LPS‐induced gp91^phox^ expression was through inhibition of AMPK activation (p‐AMPK).

## Discussion

**Figure 6:**
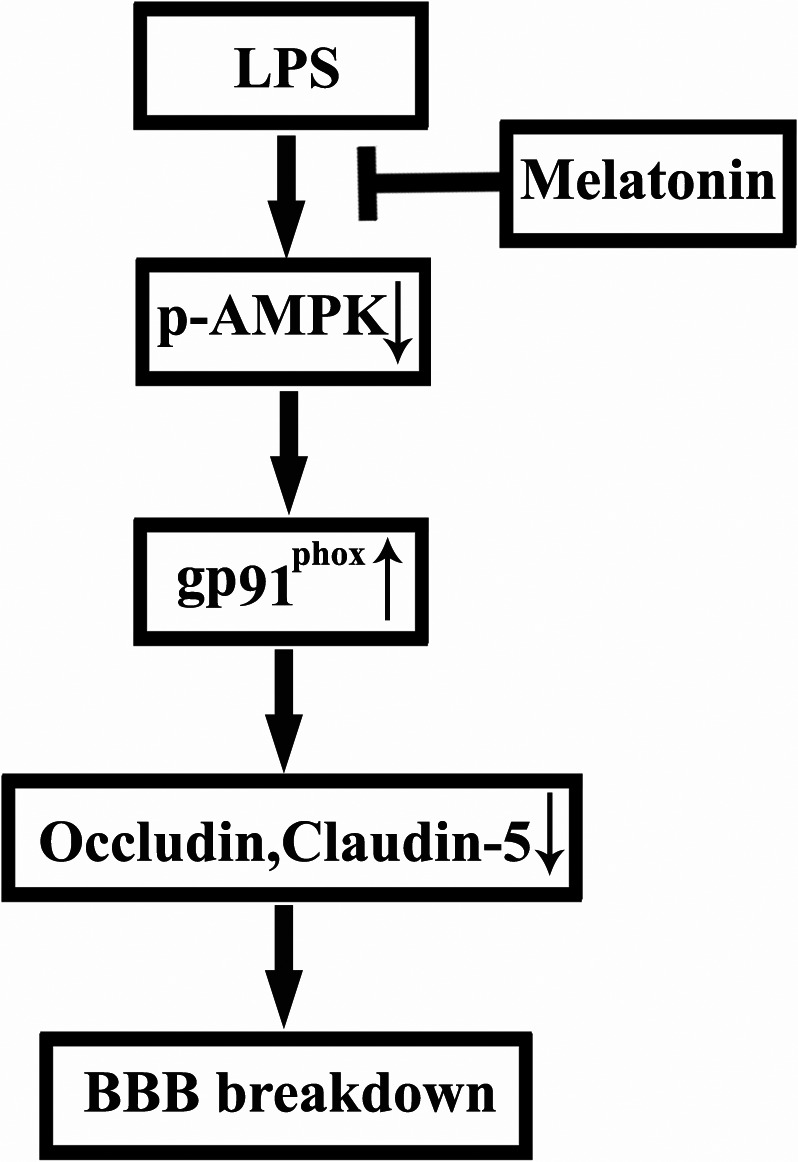
A schema for proposed molecular mechanism underlying melatonin's protective effect on LPS‐induced BBB damage in old mice. LPS‐induced BBB disruption accompanied by tight junction protein occludin and claudin‐5 degradation through decreasing AMPK activation (p‐AMPK) and upregulating gp91^phox^ protein levels. Melatonin supplementation alleviates LPS‐induced BBB damage via activating AMPK and downregulating gp91^phox^.

BBB damage induced by extrinsic or intrinsic stimuli plays an important role in neurological diseases (Rosenberg, 2012), and BBB disruption could occur in old people even when facing common stress. It is well known that the old people have decreased levels of melatonin in the brain and circulation (Karasek, 2004); however, whether there is a link between BBB disruption and decreased melatonin remains unknown. Using old mice, we investigated the effect of melatonin on LPS‐induced BBB damage. Our important findings include the following: (1) Melatonin alleviates LPS‐induced BBB damage in old mice, which is accompanied by decreased tight junction protein (TJP) degradation; (2) melatonin decreased LPS‐induced BBB damage by activating AMPK and inhibiting gp91^phox^ expression upregulation; and (3) activating AMPK by metformin significantly inhibited LPS‐induced upregulation of gp91^phox^ (Fig. 6).

LPS has been shown to dose‐dependently increase BBB permeability (Nonaka *et al*., 2004) and 1 mg kg^−1^ LPS compromised BBB integrity in young mice (Ruiz‐Valdepenas *et al*., 2011; Zhou *et al*., 2014a). Unexpectedly, we found that only 1 mg kg^−1^ LPS significantly increased the extravasation of EB and IgG in old mice, suggesting that the BBB of old mice does not appear to be more vulnerable in response to LPS than young mice.

Oxygen radical detoxification processes decrease during aging (Reiter, 1995) and there was a marked drop in pineal biosynthetic activity in aging hamster (Reiter *et al*., 1980) as well as extrapineal melatonin decreases in aging mice (Hill *et al*., 2013). Interestingly, LPS not only induces ROS production, but also induces inflammatory response and chronic melatonin treatment has shown reduction of age‐dependent inflammatory process in senescence‐accelerated mice (Rodriguez *et al*., 2007). Moreover, melatonin protects against LPS‐induced toxicity not only to the brain (Wong *et al*., 2014; Carloni *et al*., 2016), but also to other organs including myocardial hypertrophy (Lu *et al*., 2015), liver damage (Wang *et al*., 2007), and acute lung inflammation (Lee *et al*., 2009). Of note, melatonin has been shown to be protective against BBB damage induced by various stimuli, including excitotoxic injury in neonatal rats (Moretti *et al*., 2015) and transient focal cerebral ischemia in mice (Chen *et al*., 2006a,b). However, it is not clear whether melatonin decreases LPS‐induced BBB damage in old mice. Our data here clearly show that LPS‐induced BBB dysfunction is attenuated by melatonin, suggesting that pretreatment with melatonin might render the BBB of old mice more resistant to LPS stimuli. Along with BBB disruption, TJPs claudin‐5 and occludin are also degraded in LPS‐challenged old mice which is consistent with previous results obtained from *in vitro* cultured endothelial cells (Zhao *et al*., 2014) as well as in young mice (Zhou *et al*., 2014a). Of note, melatonin supplementation significantly inhibits LPS‐induced TJP degradation in old mice.

LPS has been shown to enhance oxidative injuries via promoting enzymatic ROS generation (Zhao *et al*., 2014), and NADPH oxidase is a major source of ROS generation in the brain which contributes to BBB disruption under various conditions (Chrissobolis & Faraci, 2008). NADPH oxidase has several family members, of which gp91^phox^ is the catalytic subunit. Gp91^phox^ knockout mice showed significantly less BBB damage than wild‐type mice after stroke (Kahles *et al*., 2007) and reduction of gp91^phox^ expression has shown protective effect on ischemia‐induced brain injury and BBB damage (Liu *et al*., 2008; Tang *et al*., 2010). Here, our data also show that gp91^phox^ protein levels are significantly elevated in LPS‐challenged old mice, implicating a role of gp91^phox^‐containing NADPH oxidase in LPS‐induced BBB disruption. Our findings that pretreatment with melatonin inhibits LPS‐induced gp91^phox^ upregulation indicate that melatonin may protect the BBB in LPS‐challenged old mice through reducing gp91^phox^ expression. It is worth pointing out that as the role of NADPH oxidase in BBB disruption has been well established in previous studies, here we did not use pharmacological NADPH oxidase inhibitors or genetic approach (knockout) to identify the contribution of gp91^phox^ in BBB disruption in LPS‐treated old mice. In addition, besides decreasing ROS generation through inhibiting LPS‐induced gp91^phox^ upregulation, melatonin may also function as a ROS scavenger to protect the BBB from impairment induced by LPS

AMPK is involved in melatonin‐induced modulation of endoplasmic reticulum stress and autophagy modulation after fatty liver graft preservation (Zaouali *et al*., 2013). Moreover, activation of AMPK has been shown to alleviate LPS‐induced BBB disruption (Zhao *et al*., 2014), high glucose‐induced TJP dysfunction in brain endothelial cells (Liu *et al*., 2012), and LPS‐induced ROS generation. Here, our data show that melatonin supplementation concurrently suppresses LPS‐induced AMPK inhibition and gp91^phox^ upregulation, implying that the protective effects of melatonin on TJPs may result from AMPK activation‐induced inhibition of gp91^phox^ upregulation.

Lastly, our data that activation of AMPK by metformin inhibits LPS‐induced gp91^phox^ upregulation indicate that LPS increases gp91^phox^ through regulating AMPK. This result is consistent with previous reports in which AMPK has been shown to regulate NADPH oxidase (Song & Zou, 2011; Ou *et al*., 2013; Tang *et al*., 2014). As an example, AMPKα2 has been reported to function as a physiological suppressor of NADPH oxidase and ROS production in endothelial cells, and its deletion causes aberrant expression and activation of NADPH oxidase and consequent endothelial dysfunction *in vivo* (Wang *et al*., 2010). In addition, AMPK activation ameliorates oxidative stress by suppressing NADPH oxidase‐derived ROS production in endothelial cells (Wang *et al*., 2010; Song *et al*., 2011).

In summary, our results demonstrate that LPS induces BBB disruption, AMPK inhibition and gp91^phox^ upregulation in old mice, and melatonin supplementation alleviates LPS‐induced BBB damage via activating AMPK and downregulating gp91^phox^, supporting that decreased levels of melatonin may contribute to common stress‐induced BBB disruption in old people.

## Experimental procedures

### Animals

Twenty‐four to 28‐month‐old C57BL/6 mice were purchased from Tongji University Experimental Animal Center (Shanghai, China). They were housed 4–5 per cage under constant temperature (23 ± 1°C) and light‐controlled vivarium (12‐h light/12‐h dark cycle). Mice that housed in the same cage underwent the same treatment. Food and water were available *ad libitum*. All animal procedures were approved by the University Committee on Animal Care of Soochow University and performed according to the NIH Guide for the Care and Use of Laboratory Animals. All efforts were made to reduce the number of animals and to minimize animal suffering. Detailed number of animal use for each experiment was listed in Figure legends.

### Tissue processing and drug administration

Mice were anesthetized with an overdose of chloral hydrate (60 mg kg^−1^, i.p.), followed by transcardially perfusion under deep anesthesia with cold PBS to remove intravascular blood. Brain was quickly removed and stored at −80°C until further use. The brain perfused 4% paraformaldehyde (PFA) was postfixed in 4% PFA at 4°C for 2 h and then stored in a 10% sucrose solution at 4°C until further experiment.

Melatonin (Sigma, St Louis, MO, USA) was dissolved in 2% ethanol (diluted in saline) (Rennie *et al*., 2008), given intraperitoneally (i.p.) at 9:00 every 24 h at a dose of 10 mg kg^−1^ day^−1^ for 1 week (Chern *et al*., 2012; Zhou *et al*., 2014a). LPS (Sigma) was dissolved in saline and administered i.p. at a dose of 1 mg kg^−1^ (Ruiz‐Valdepenas *et al*., 2011; Zhou *et al*., 2014a) at 24 h after the last melatonin injection (Ortiz *et al*., 1999).

### Assessment of Evan's blue (EB) dye leakage

BBB damage was determined by assessing the extravasation of EB (Sigma). Twenty‐four hours after LPS treatment, EB (2% wt/vol in sterile PBS) was administered (3 mL kg^−1^) through the tail vein, and 20 min later, mice were transcardially perfused with ice‐cold PBS and then the brain was quickly taken out and BBB disruption was quantitatively assessed by measuring EB contents as we have reported (Liu *et al*., 2016). In brief, the brain tissue was harvested as described above and homogenized in 50% wt/vol trichloroacetic acid (Sigma). After centrifugation (14 000 g for 15 min) at 4°C, the supernatant was collected, and fluorescence intensity (μg mL^−1^) was measured at 620 nm on a microplate fluorescence reader (Infinite M200 Pro; TECAN, Grodig, Austria). The total Evan's blue content (μg) in each sample was derived from concentrations of external standards (1–20 μg mL^−1^). The difference of dye content between each group was calculated as Evan's blue leakage and expressed as per gram of brain tissue (μg g^−1^).

### Evaluation of BBB permeability by detection of immunoglobulin G (IgG) leakage

IgG leakage was used to evaluate BBB permeability as we described previously (Wang *et al*., 2016). In brief, the 20‐μm‐thick section was fixed with 4% PFA for 20 min at room temperature, followed by staining with Cy‐3‐conjugated affinity pure goat anti‐mouse IgG (1:200; KPL, Gaithersburg, MD, USA) for 2 h. After mounted with a glass coverslip, the slide was scanned in a LSM 700 microscope (Carl Zeiss) and the coronal image was reconstructed using Adobe Photoshop.

### Immunostaining for RECA‐1, cleaved caspase‐3, occludin, and claudin‐5

The 20‐μm‐thick cryosection was fixed with 4% PFA for further analysis as we described previously (Wang *et al*., 2016). In brief, blocking buffer containing 0.3% Triton X‐100, 1% BSA, and 5% goat serum was used to block nonspecific binding. Sections were then incubated overnight with anti‐RECA‐1 (1:200 dilution; Abcam, Cambridge, UK), anticleaved caspase‐3 (1:300 dilution; R&D System Inc., Minneapolis, MN, USA), anti‐occludin (1:100 dilution; Invitrogen, Carlsbad, MA, USA), anti‐claudin‐5 (1:200 dilution; Invitrogen) primary antibody at 4°C. After 24 h, sections were incubated with mouse (1:1000 dilution; Boster, Wuhan, Hubei, China) secondary antibody for 2 h at room temperature. Confocal images were obtained using a laser scanning confocal microscope (Zeiss LSM 700, Carl Zeiss).

### Cell culture

Mouse brain microvascular endothelial cells bEND3 (American Type Culture Collection, Rockville, MD, USA) were grown as a monolayer in DMEM with 15% fetal bovine serum (FBS), 100 U mL^−1^ penicillin, and 100 μg mL^−1^ streptomycin at 37°C in a humidified incubators with 5% CO_2_ and 95% room air. Cells were subcultured into 35‐mm dishes and allowed to grow to confluence before incubating with metformin.

The confluent endothelial cells were first treated with metformin (Beyotime, Shanghai, China) at a dose of 0, 0.1, 0.5, and 1 mm for 24 h to define the optimal dose in activating AMPK. Then, the cells were treated with metformin at the optimal dose for 8 h before adding 1 μg mL^−1^ LPS (Zhao *et al*., 2014), and 16 h later, Western blot was used to detect gp91^phox^ protein levels.

### Western blot analysis for p‐AMPK, AMPK, occludin, claudin‐5, and gp91^phox^

Homogenate aliquots (50 μg of total protein) were boiled and then electrophoresed in 12% SDS‐PAGE acrylamide gels, transferred onto PVDF membrane (Millipore, Billerica, MA, USA), and incubated for 1 h in TBS‐T (Tris‐buffered saline and 0.1% Tween‐20) containing 5% nonfat milk. Membranes were then incubated with primary antibodies against p‐AMPK (1:1000; Cell Signaling Technology, Danvers, MA, USA), AMPK (1:1000; Cell Signaling Technology), gp91^phox^ (1:1000; BD Transduction Laboratories, Lexington, KY, USA), occludin (1:300; Invitrogen), or claudin‐5 (1:500; Invitrogen) overnight at 4°C, washed in TBS‐T, and then incubated for 1 h at room temperature with corresponding HRP‐conjugated anti‐rabbit or anti‐mouse antibodies (1:2000; Boster). The membranes were developed with the SuperSignal West Pico HRP substrate kit (Pierce, Rockford, IL, USA) and photographed. Protein band intensities were quantitated after normalization to beta actin.

### Statistical analysis

The data are presented as mean ± SEM. Statistical analysis was carried out using ANOVA (SPSS software, version 17.0). Significant effects were probed using Newman–Keuls *post hoc* comparison. A value of *P *<* *0.05 was considered statistically significant.

## Funding info

This work was supported by Soochow University Research startup fund (Q421500113), by National Natural Science Foundation of China (31271371, 81371328, 81571149, 81671145), by Natural Science Foundation of Jiangsu Province of China (L221506415), and by a grant from Shenzhen Science and Technology Innovation Commission (KQCX20140521101427034). This work was also partly supported by Priority Academic Program Development of Jiangsu Higher Education Institutions of China.

## Conflicts of interest

The authors declare that they have no conflict of interests.

## Author contributions

JL, WL, and XJ are the principal investigators at the three collaborating institutions and are responsible for project design, supervision of technical personnel, interpretation of results, and preparation of manuscript drafts. YES and CFL provided advice on experimental design and interpretation, and comments on the manuscript. XW, GX, WCL, HS, MW, YS, and XL performed experiments, analyzed the data, made the figures, and drafted the manuscript.
